# Factors associated with childhood chronic malnutrition in West and Central Africa: a scoping review

**DOI:** 10.11604/pamj.2022.43.45.32820

**Published:** 2022-09-28

**Authors:** Pengdewende Maurice Sawadogo, Drissa Sia, Eric Tchouaket Nguemeleu, Jean-François Kobiane, Yentema Onadja, Stephanie Robins

**Affiliations:** 1 Institut Supérieur des Sciences de la Population (ISSP), Université Joseph Ki-Zerbo, Ouagadougou, Burkina Faso,; 2 Department of Nursing, Université du Québec en Outaouais, Saint-Jérôme, Québec, Canada,; 3 Département de Médecine Sociale et préventive, École de Santé Publique, Université de Montréal, Montréal, Québec, Canada; 4 Département de Gestion, d’Evaluation et de Politique de Santé, École de Santé Publique, Université de Montréal, Montréal, Québec, Canada

**Keywords:** Chronic malnutrition, child, factors associated, West Africa, Central Africa

## Abstract

Chronic malnutrition is a major public health concern that is the focus of a large body of scientific research. However, there is no synthesis of knowledge about the factors associated with this disease in West and Central Africa, where its prevalence is particularly high. We conducted a systematic search for scientific articles published between January 1^st^, 2000, and October 15^th^, 2020, that focus on chronic malnutrition in children in West and Central Africa. We queried CAIRN, PubMed, CINAHL, MEDLINE, Scopus, and Google Scholar databases for this purpose. The search process followed the recommendations of Arksey and O'Malley. Items reported in this review follow the PRISMA-ScR guidelines. Sixty articles involving children from a total of twenty (20) countries, mainly Ghana and Nigeria, were included in the final analysis. The data used were predominantly cross-sectional and were mainly drawn from demographic and health surveys. The analysis revealed that chronic malnutrition in children is associated with sociocultural, economic, and healthcare factors related to the characteristics of children, mothers, households, and communities. The association with children's vulnerability to disease, maternal education, purchasing power, and autonomy need to be further investigated in West and Central Africa. Further analysis using longitudinal data is also needed to better understand the factors associated with chronic malnutrition in West and Central Africa.

## Introduction

Chronic malnutrition in children is a growth disorder resulting from a long-term deficiency in nutrient intake. The condition brings numerous adverse consequences to health and economic status which are long-lasting and sometimes irreversible [1]. Studies have shown that women who suffered from chronic malnutrition in childhood are more predisposed to complications during childbirth [2,3]. There is also evidence that children who suffered from chronic malnutrition have an increased risk of obesity, high blood pressure, and renal failure in adulthood [3-5]. In addition, chronic malnutrition is associated with reduced physical and intellectual capacity resulting from a long-term deficiency of iodine and iron, an effect that is often irreversible after the child reaches the age of two [6,7]. This reduced cognitive capacity of children -who should become future workers- demonstrates how chronic malnutrition handicaps the development of countries. As such, it is considered “a symptom of past deprivation and a prediction of future poverty” [8].

In sub-Saharan Africa, chronic malnutrition is widespread, affecting one-third of children under the age of five [9]. Evidence-based scientific research will be required to support decision-making to tackle this high level of chronic malnutrition and successfully achieve the goal of eliminating it by 2030, following the Sustainable Development Goals [10].

Given the importance of the subject, several empirical studies have analyzed factors contributing to childhood chronic malnutrition. There are two review articles concerning the whole of sub-Saharan Africa [11,12], which covers all of West and Central Africa. However, one of them does not cover the last four years (2018, 2019, 2020 and 2021) [11]. Also, the most recent review only took into account nationally representative studies [12]. It, therefore, excluded the many studies conducted at the sub-national level. Other research has examined the effect of maternal education on chronic malnutrition in children [13].

To date, there is no article synthesizing knowledge on the factors associated with chronic malnutrition in West and Central Africa, which is the most affected sub-region [9]. The specific health and socio-cultural characteristics of this sub-region support the need for such knowledge synthesis. This article addresses the need for a synthesis of knowledge on the factors associated with chronic malnutrition in West and Central Africa. It is designed as a scoping review, which is an alternative to a classic systematic review when the literature on a subject is extensive [14], which is the case for chronic malnutrition in this setting.

## Methods

**Study design:** this scoping review was conducted according to the framework defined by Arksey and O'Malley [14] which is well established and frequently used [15]. The review was written following the recommendations of Preferred Reporting Items for Systematic reviews and Meta-Analyses extension for Scoping Reviews (PRISMA-ScR) [16].

**Protocol registration:** there has been no protocol and registration of this scoping review.

**Research question:** the scoping review was conducted around the following question: “according to the scientific literature, what are the factors associated with chronic malnutrition in children in West and Central Africa?”.

**Search strategy and data source:** the search question was analyzed to identify keywords representing population, intervention, comparators, outcomes, timing, and study design (PICOTS), which were then used to build search strategies. These search strategies were then applied to the CAIRN, PubMed, CINAHL, MEDLINE, Scopus, and Google Scholar databases. The keyword searches used included free text and controlled vocabulary. Orthographic variants and synonyms of the keywords were also taken into consideration in the construction of the search equations. These equations enabled a systematic selection of all scientific articles that contained these words in their title, abstract, or keywords. The search equation used was: (“chronic malnutrition” or “growth disorder” or “stunted growth” or stunting or “nutritional status” or undernutrition or malnutrition) and (child* or infant* or under-five or preschool).

**Eligibility criteria:** eligibility criteria, both inclusion and exclusion, were defined to guide the selection of articles.

**Inclusion criteria:** all articles included in this scoping review met the following criteria: i) the study concerned one or more countries in West Africa (Benin, Burkina Faso, Central African Republic, Chad, Ivory Coast, Democratic Republic of Congo, Gambia, Ghana, Guinea, Guinea-Bissau, Liberia, Mali, Mauritania, Niger, Nigeria, Senegal, and Sierra Leone) or Central Africa (Angola, Cameroon, Gabon, Equatorial Guinea, Republic of Congo, Sao Tome and Principe); ii) the study was published between January 1, 2000, and October 15, 2021; iii) the study addressed chronic malnutrition assessed by a height/age ratio; iv) the measurement of height respected the World Health Organization (WHO) standard of measuring height in the supine position for children under two and in the standing position for those aged two and over according to WHO and UNICEF [17]; v) the reference standard used for estimating the nutritional status of children was either the National Center for Health Statistics (NCHS) standard [18] or the new WHO standard [19]; vi) the study assessed the factors associated with chronic malnutrition in children under five years of age; vii) finally, the article was published in French or English.

**Exclusion criteria:** although meeting the inclusion criteria described above, some articles were excluded for the following reasons: i) the articles were restricted to specific populations such as premature infants, children who were hospitalized or attending a given health service, or children suffering from a particular disease or condition (HIV, congenital pathology, etc.); ii) the articles were methodological guides or handbooks; iii) the data used were collected before January 1, 2000.

**Figure 1:**
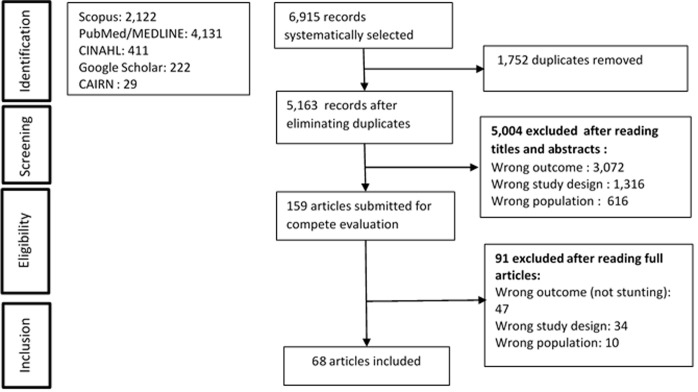
flow chart showing the process for selecting the articles

**Selection process:** the selection of articles was carried out in several steps (Figure 1). It began with the automatic selection of articles by applying search strategies in all databases. References were then exported to Endnote where perfect duplicates were eliminated. Once the duplicates were removed, the remaining references were then exported to the RAYYAN website and screened according to the eligibility criteria. The first screening evaluated the articles by reading titles and abstracts. Following this, the full text of the selected articles was read for final inclusion. Two authors (Pengdewende Maurice Sawadogo and Drissa Sia) separately selected the references by reading the titles and abstracts. The references for which there was a discordance were secondarily examined by a third author (ET). The same process was observed for the complete reading of the articles.

**Data extraction from included studies:** the data were extracted using a grid designed by the authors. The extraction of data from the first eight (8) articles was done by the whole team of authors during a working meeting. The remaining articles were distributed among the authors for extraction.

**Collating, summarizing and reporting the findings:** the data extracted from the included articles were designed following the grid. The relevant information obtained includes references of the article, country, age of children, types of study and methods of analysis, and significant risk factors. Subsequently, these data were organized by country and by geographical area.

## Results

Applying our search strategy identified a total of 6,915 articles: 2,122 in SCOPUS, 4,131 in PubMed/MEDLINE, 411 in CINAHL, 222 in Google Scholar, and 29 in CAIRN. There were 1,752 duplicates that were removed. Of the 5,163 remaining articles, 4,978 (96.4%) were excluded after reading their titles and abstracts. Also, 40 articles were initially classified as “undecided”, they were then re-evaluated, and 26 were excluded and 14 were retained. The 159 articles retained at this stage were read in their entirety and analyzed according to see if they met eligibility criteria. In the end, 68 articles were included. The entire selection process is presented in Figure 1.

**Characteristics of the selected studies:** four studies [20-23] were published in French and 64 [24-87] in English. The classification of the included articles by country shows that nearly half were conducted in two English-speaking countries, namely Ghana (n=19) and Nigeria (n=13). In French-speaking countries, most studies were conducted in Burkina Faso (n=12), Senegal (n=6), and Democratic Republic of Congo (DRC) (n=4). No study concerning Togo, Cape Verde, Sierra Leone, and Sao Tome and Principe was included.

**Methods for identifying factors associated with chronic child malnutrition:** the characteristics of the selected studies, including the methods for analyzing the factors associated with chronic child malnutrition, are presented in Table 1, Table 1 (suite), Table 2, Table 2 (suite) and Table 3. The main sources of data were demographic and health surveys (DHS) or multiple indicator cluster surveys (MICS), which were used in 31 studies. Overall there were 63 cross-sectional [20-45,47-55,57-60,62-70,72-84,86,87] and 5 longitudinal study designs [46,56,61,71,85]. Child growth references of the NCHS standard were used in eight studies [22,23,41,54,56,58,68,81]. The new WHO standard was used in the other 60 studies [20,21,24-40,42-53,55,57,59-67,69-80,82-87].

**Table 1:** characteristics of selected studies in Ghana and Nigeria

References	Country	Age of children (months)	Types of study and methods of analysis	Factors associated with chronic malnutrition
Aheto JMK, 2020	Ghana	0-59	Cros-sect (DHS), lin reg	Child age decreasing, child ale sex, twinship, having diarrhoea, delivering at home, small size at birth; maternal age decreasing, number of children under five increase, household not having health insurance, poor wealth status, no maternal education
Anin *et al*. 2020	Ghana	6-23	Cros-sect; log reg	Child age ≥12 months, low iron-rich food intake, mother's height below 160cm
Appiah *et al*. 2021	Ghana	6-24	Cros-sect, multinomial log reg	Child not being fed from separate bowls, initiation of complementary feeding, before the child had six months
Groot *et al*. 2020	Ghana	6-59	Cros-sect; lin reg	Low household income, low vaccination coverage, not sleeping under bednets, low maternal education
Essilfie *et al*. 2020	Ghana	0-59	Cros-sect (DHS), quant reg	Child male sex, household not owning radio, low maternal education, maternal having a manual job, maternal low autonomy in decision making, mother experiencing domestic violence, small household size, long time for getting water
Wemakor *et al*. 2018	Ghana	6-59	Cros-sect; log reg	Maternal age under 19
Ali *et al*. 2017	Ghana	6-59	Cros-sect; log reg	Child's age >12 months, male sex, mother's height <150 cm
Wemakor *et al*. 2016	Ghana	0-59	Cros-sect; log reg	Maternal depression, low SES
Boah *et al*. 2019	Ghana	0-59	Cros-sect (DHS), log reg	Child's age ≥12 months, birth rank ≥5, birth weight (≤2.5 kg), maternal low autonomy, region
Saaka *et al*. 2015	Ghana	6-23	Cros-sect; Log Reg	Children who started supplemental feeding at six months were less likely to be stunted than those who started earlier or later
Frempong *et al*. 2017	Ghana	0-59	Cros-sect (MICS); lin reg	Child's age increasing, (IYCF) score, frequency of fever, child being sibling, maternal age increasing, ethnicity, low household SES, region, rural residence
Saaka M., 2014	Ghana	0-36	Cros-sect; lin reg	Maternal nutritional knowledge
Ewusie *et al*. 2017	Ghana	0-59	Cros-sect; log reg	Child age ≥12 months, male sex, no/low maternal education, low household SES
Aheto *et al*. 2015	Ghana	0-59	Cros-sect (DHS) mlev lin reg	Child's age increasing, breastfeeding duration decreasing, small birth size, twinship, lack of maternal health insurance and low maternal BMI, low household SES
Annim *et al*. 2015	Ghana	0-59	Cros-sect (DHS); lin reg	Child's age increasing, small birth size, not-breastfeeding, Maternal age increasing, household number of children under 16 increasing, low SES, region
Darteh *et al*. 2014	Ghana	0-59	Cros-sect (DHS); log reg	Child's age ≥6 months, mother's age <25 years, low household SES, number of children in household >4, region
Nikoi *et al*. 2013	Ghana	0-59	Cros-sect (DHS) mlev lin reg	Child's age increasing, non-vaccination, small birth size, duration of breastfeeding, low household SES, population density
Kang *et al*. 2013	Ghana	3-23	Long, log reg (propensity score)	Frequency of diarrhoea in child
Hong R., 2007	Ghana	0-59	Cros-sect (DHS); log reg	Child age ≥12 months, male sex, birth weight <2.5 kg, feeding frequency <2/day, non-vaccination, maternal non-education, low household SES, region
Obayelu and Adeleye	Nigeria	8-59	Cros-sect (DHS); prob reg	Child male sex, child not completing vaccination, mother having a farming job, source of drinking water unprotected low household income

MICS: multiple indicator cluster surveys; IYCF: infant and young children feeding practices; long: longitudinal; cros sect: cross-sectional; log reg: logistic regression; lin reg: linear regression; prob reg: probit regression; Mlev: multilevel; DHS: demographic and health survey, SES: socio-economic status

**Table 1(suite):** characteristics of selected studies in Ghana and Nigeria

References	Country	Age of children (months)	Types of study and methods of analysis	Factors associated with chronic malnutrition
Fadare *et al*. 2019	Nigeria	6-23	Cros-sect (DHS) lin reg	Child's age increasing, male sex, being a twin, low birth weight, maternal nutritional knowledge, age at first birth, household number of children <5, low SES, region
Fadare *et al*. 2019	Nigeria	6-59	Cros-sect; log reg	Child being sick, low maternal education no-consumption of micronutrient-rich foods, no ownership of animals and assets (refrigerator), household size increasing
Agu *et al*. 2019	Nigeria	3-24	Cros-sect (DHS); log reg	Child's male sex, Mothers not practicing exclusive breastfeeding, domestic violence against mothers, mother's lack of education through high school, mother's not being married, Peulh, Hausa, and Igbo ethnic groups
Amare *et al*. 2018	Nigeria	6-23	Cros-sect (DHS); log reg	Child's age increasing, male sex, no vitamin A supplementation, having diarrhoea, maternal age at first birth, BMI, household has no radio, no diversification of diet, Muslim religion
Nwosu *et al*. 2018	Nigeria	0-59	Cros-sect (DHS); prob reg	Child's age increasing, male sex, low maternal education, no vitamin A supplementation, household income, no toilet use
Akombi *et al*. 2017	Nigeria	0-59	Cros-sect (DHS) mlev log reg	Child age ≥12 months, male sex small birth size, diarrhea, breastfeeding duration <12 months, mother's occupation, duration of breastfeeding, mothers not being assisted during childbirth, household low SES (poor or very poor), geographic area
Udoh *et al*. 2016	Nigeria	6-11	Cros-sect; LOG REG	Child has inadequate meal diversification, inadequate meal frequency, maternal illness during the previous month
Balogun *et al*. 2015	Nigeria	0-59	Cros-sect; log reg	Child's pneumonia, not vaccinated for measles, DPT2, paternal low education
Adekanmbi *et al*. 2013	Nigeria	0-59	Cros-sect (DHS) mlev log reg	Individual factors: child's age ≥11 months, male sex, twinship, low birth weight (LBW), low maternal education, maternal BMI <18.5 or ≥25, low SES, small intergenesic period, care-seeking behaviour, contextual factors: low education rate, region
Ajao *et al*. 2010	Nigeria	0-59	Cros-sect; log reg	Poor maternal nutrition, low maternal education,
Uthman OA, 2009	Nigeria	0-59	Cros-sect (DHS) mlev log reg	Short breastfeeding duration, low household SES, low health service attendance score (e.g. antenatal care, delivery, immunization)

NB:* data not available in the articles consulted; long: longitudinal; cros sect: cross-sectional; log reg: logistic regression; lin reg: linear regression; prob reg: probit regression; Mlev: multilevel; DHS: demographic and health survey, SES: socio-economic status; DPT: diphtheria, pertussis, and tetanus

**Table 2:** characteristics of selected studies in West Africa, excluding Ghana and Nigeria

References	Country	Age of children (months)	Types of study and methods of analysis	Factors associated with chronic malnutrition
Agbota *et al*. 2020	Benin	12	Long; log reg	Maternal body mass index (BMI) <18.5 before conception
Alaofè *et al*. 2017	Benin	6-59	Cros-sect; lin reg	Maternal mobility (permission to go to the market, to the health center or to see friends), leadership
Aké-Tano *et al*. 2010	Ivory Coast	0-59	Cros-sect; log reg	Child stopping breastfeeding before age of 2, frequency of diarrhoea and fever, maternal BMI <18.5
Mank *et al*. 2020	Burkina Faso	8-59	Cros-sect; lin reg	No diversification of child's meals (no consumption of dairy, vegetables, and fruits)
Gelli *et al*. 2019	Burkina Faso	6-59	Cros-sect; mlev log reg	Child's age increasing, male sex, borehole water supply, child not being clean, low maternal education, low household income
Sié *et al*. 2018	Burkina Faso	6-59	Cros-sect, log and lin reg	Low dietary diversity score
Bougma *et al*. 2019	Burkina Faso	6-59	Cros-sect; log reg	Child's age 12-23 months, birth rank >3, birth weight <2500 g, mother delivering at home, mother's lack of occupation, low household SES, household size ≥10, residence outside the city where the healthcare is located
Chuang *et al*. 2020	Burkina Faso	0-59	Global Moran's I test; generalized estimation equation	Low measles vaccination coverage; low agricultural land coverage; low sanitation coverage (toilets)
Adeyemi *et al*. 2019	Burkina Faso	0-59	Cros-sect (DHS) multivariate conditional autoregressive model	Child's age <12 months, female sex, frequency of diarrhoea low household SES, lack of access to electricity
Poda *et al*. 2017	Burkina Faso	0-59	Cros-sect (DHS); log reg	Age ≥12 months, male sex, twinship, small birth size, siblings ≥3, maternal age ≥35; maternal BMI <18.5 maternal non-education, maternal marital status, low household SES
Fregonese *et al*. 2017	Burkina Faso	12-59	Long; generalized structural equation modeling	Environmental contamination index (water, sanitation, hygiene, yard cleanliness, and proximity to animals)
Prado *et al*. 2019	Burkina Faso	18	Long; structural equation modeling	Short maternal height, low BMI, low education; household unimproved source of drinking water; low dietary diversity
Sawadogo *et al*. 2006	Burkina Faso	6-35	Cros-sect; lin reg	Low index of feeding practices
Makamto *et al*. 2018	Mali	6-24	Cros-sect; log reg	Child's age ≥12 months, male sex, low meal diversity score, frequency of diarrhoea; low household SES, region
Berenger *et al*. 2019	Senegal	0-59	Cros-sect (DHS) Mlev log reg	Individuals: child's age >12 months, male sex, intergenesic period <24 months, recent illness (fever, diarrhoea, cold), low birth weight, low maternal education, low household SES; contextual: lack of use of appropriate toilets, low decision-making power of women in the district, lack of access and use of maternal health services, region

NB: *data not available in the articles consulted; long: longitudinal; cros sect: cross sectional; log reg: logistic regression; lin reg: linear regression; prob reg: probit regression; mlev: multilevel; DHS: Demographic and health survey; SES: socio-economic status; BMI: body mass index

**Table 2(suite):** characteristics of selected studies in West Africa, excluding Ghana and Nigeria

References	Country	Age of children (months)	Types of study and methods of analysis	Factors associated with chronic malnutrition
Buttarelli *et al*. 2013	Senegal	0-36	Cros-sect; log reg	Child's age ≥24 months; low birth weight
Bork *et al*. 2012	Senegal	6-36	Long; mixed model	Low dietary diversity index and low food variety index in children aged 6-24 months
Ntab *et al*. 2005	Senegal	8-42	Cros-sect; lin reg	No relationship between dietary practices and stature growth
Gupta *et al*. 2017	Senegal	6-23	Cros-sect; log reg	Child's age increasing; unsafe source of drinking water
Woodruff *et al*. 2018	Guinea	0-59	Cros-sect (DHS); log reg	0-5 months: child male sex, twinship, no maternal vitamin A supplementation; 6-23 months: child anemia, birth size, twinship, child age increasing, maternal low BMI, low height, low household SES; 24-59 months: child male sex; siblings, child having anemia, child not continuing breastfeeding, maternal anemia, maternal short in height, not-taking vitamins, household managing children's faeces inappropriately, unsafe drinking water, high household dependency ratio (≥ 1.5)
Sobkoviak *et al*. 2012	Liberia	0-59	Cros-sect (DHS); log reg	Child age ≥12 months, domestic sexual violence to mother, region

NB: * data not available in the articles consulted; long: longitudinal; cros sect: cross-sectional; log reg: logistic regression; lin reg: linear regression; prob reg: probit regression; mlev: multilevel; DHS: demographic and health survey, SES: socio-economic status; BMI: body mass index

**Table 3:** characteristics of selected studies in a country in Central Africa or including several countries

References	Country	Age of children (months)	Types of study and methods of analysis	Factors associated with chronic malnutrition
Humbwavali *et al*. 2019	Angola	0-23	Cross-sectional; poisson regression	Frequency of diarrhea in child, maternal occupation
Fernandes *et al*. 2017	Angola	0-59	Cross-sectional; poisson regression	Child's age <12 months, child male sex, intestinal parasitosis, ear infections, household source of drinking water
Dapi *et al*. 2019	Cameroon	0-59	Cross-sectional; logistic regression	Child male sex, 1st and 2nd rank of birth, mother being a farmer
Thorne *et al*. 2013	Guinea-Bissau	0-59	Cross-sectional; logistic regression	Child's age increasing, child having anemia, rural residence
Custodio *et al*. 2008	Equatorial Guinea	0-59	Cross-sectional; logistic regression	Child's age ≥12 months, household with no fisherman, not in proximity to a hospital
Vonaesch *et al*. 2017	CAR	0-59	Cross-sectional; logistic regression	Child's age; child's sex
Kobelembi *et al*. 2004	CAR	0-59	Cross-sectional (MICS); logistic regression	Child's age ≥12 months, male sex, low maternal education, maternal occupation, young maternal age, ethnicity; household low socioeconomic status (SES), region
Kismul *et al*. 2017	DRC	0-59	Cross-sectional (DHS); logistic regression	Child's age ≥12 months, male sex, intergenesic interval < 24 months, early breastfeeding initiation, young maternal age (< 20 years), maternal height, household low SES, region
Mukalay *et al*. 2010	DRC	0-59	Cross-sectional; logistic regression	Child's male sex, age >12 months, low maternal education, household with no water tap
Wit *et al*.	Burkina Faso, Mali	0-59	Cross-sectional; logistic regression	Child male sex, child age > 12 month, high incidence of malaria
Amugsi *et al*. 2017	Ghana, Nigeria, RDC	6-59	Cros-sect (DHS); multivariate quantile regression	Low dietary diversity score, male sex, increasing age, low SES, small household size
Garenne M. 2018	5 countries†	12-59	Cros-sect (DHS); lin reg	Child's age increasing, female sex, low household SES, rural residence, low maternal education
Yaya, Odusina *et al*. 2020	30 countries from SSA	0-59	Cross-sectional (DHS); logistic regression	Mothers' non-participation in decision making, mother's experiencing violence, mother holding incorrect attitudes toward domestic violence
Yaya, Oladimeji *et al*. 2020	35 countries in SSA	0-59	Cross-sectional (DHS); logistic regression	Child's age, male sex and twinship and the child's birth interval, low maternal education, polygamous unions, multiple unions; young age, the fact that the child does not live with the mother, the fact that the mother's husband lives in the household, residence in a rural area
Anand and Roy, 2016	13 countries in SSA	0-59	Cross-sectional (DHS); logistic regression	Household unsafe water source, household not having access to safe toilet
Oyekale and Oyekale, 2009	Gambia and Niger	0-59	Cross-sectional (DHS); logistic regression	Child's age increasing, breastfeeding, frequency of fever and diarrhea, no polio vaccination, being an orphan, low maternal education, low access to safe water, electricity, no possession of radio, television urbanization

NB: * data not available in the articles consulted; long: longitudinal; cros sect: cross sectional; log reg: logistic regression; lin reg: linear regression; prob reg: probit regression; DRC: Democratic Republic of Congo; CAR: Central African Republic; †: Burkina Faso, Mali, Chad, Niger, Senegal, and Mauritania

**Factors associated with chronic malnutrition in children:** the analysis of the 68 selected articles showed that child, maternal, household, and community characteristics are statistically associated with chronic malnutrition in children in West and Central Africa (Table 1, Table 1 (suite), Table 2, Table 2 (suite), Table 3).

**Child characteristics associated with chronic malnutrition:** child characteristics are those that are the most frequently associated with chronic malnutrition. These include age, sex, twinship, birth weight and height, health status, vaccination status, and dietary intake. The effects of age and sex were the most common across studies. The association with age was reported in 41 studies and indicated an increased risk of chronic malnutrition with increasing age. The effect of sex was found in 30 studies and most of them showed a higher risk in boys than in girls. The effects of twinship (n=7), birth rank (n=3), birth weight (n=6), birth height (n=3), birth interval (n=2), disease frequency (n=13), vaccination status (n=7), breastfeeding (n=3), meal diversification (n=9), meal frequency (n=2), and vitamin A and iron supplementation (n=1) were also found. However, some studies that investigated these factors found that the sex or age of the child was not associated with chronic malnutrition in children. This was the case of two studies conducted in Nigeria [38] and Burkina Faso, Mali, Chad, Senegal, and Mauritania [22] which found no effect on the age of the child. Three studies, conducted in Nigeria, Burkina Faso, and Ghana, concluded that the sex of the child was not statistically associated with his or her nutritional status [21,38,60].

**Maternal characteristics associated with childhood chronic malnutrition:** significant effects of maternal characteristics, including education, age, mobility, leadership, experience with domestic violence, decision-making autonomy, ethnicity, marital status, and health status were reported in the reviewed studies. The effect of maternal education was reported in 19 studies and showed a reduction in the risk of chronic malnutrition with an increased maternal level of education. These effects of education are particularly apparent when mothers have an education level from secondary school and up [48,49,77,84].

The effects of a mother's purchasing power, as assessed by employment status or income level, were demonstrated in six studies in Angola, Burkina Faso, Cameroon, Ghana (n=2), and Nigeria [21,30,32,57,67,80]. These studies showed that the risk of chronic malnutrition is relatively lower for children of high-income mothers. An association between maternal age and chronic malnutrition in children has also been established in nine studies. They demonstrate that the risk of chronic malnutrition was higher in children of adolescents [36] or relatively younger mothers [24,26,36,54,60,66,74,77].

The effects of maternal autonomy, including leadership, participation in decision making, experience, or attitude toward violence were highlighted in five studies. They found that children of mothers with decision-making autonomy are less likely to suffer from chronic malnutrition [30,33,41,72,79,84]. Five other studies showed an association between maternal nutritional status and child chronic malnutrition. Usually, the risk was higher for children of mothers with a nutritional deficiency (BMI <18.5) [46,81,83,85,87]. Some studies also reported a higher risk of chronic malnutrition for children of mothers of low height [27,46,78]. The effect of maternal ethnicity was reported in two studies [60,84]. In addition, four studies conducted in Burkina Faso, Ghana and Nigeria have established that children whose delivery occurred in a health center or whose mothers had prenatal consultation(s) were less likely to suffer from chronic malnutrition [21,26,38,80]. Finally, in Burkina Faso, children of mothers with a partner were less likely to suffer from chronic malnutrition than children of non-partnered mothers [47].

Some studies found that maternal and child characteristics are not statistically associated with chronic malnutrition in children. For example, four studies conducted in Ghana, Senegal, and Nigeria concluded that there was no effect of maternal education on chronic child malnutrition [39,45,70,72]. It is the same for purchasing power, in that the absence of its effect has been reported in Nigeria [39].

**Household characteristics associated with childhood chronic malnutrition:** the analysis of the articles revealed that certain household characteristics are associated with child chronic malnutrition. These characteristics include socioeconomic status, family composition, and access to water and sanitation.

The effect of socioeconomic status was found in ten studies and was assessed by the level of economic well-being [21,26,32,47,59,64,66,80,86] or livestock ownership [63]. These studies show a low propensity for chronic malnutrition in children from households with high socioeconomic status. In rare cases, as in Pikine, Senegal, the effect of household socioeconomic status was not significant [70]. Five studies reported that the household family composition was significantly associated with chronic malnutrition in children. Children from single-parent or monogamous households or those living in a household with relatively few young children were at lower risk of suffering from chronic malnutrition [24,26,47,60,66].

Eight studies conducted in Angola [62], Burkina Faso [46,59], Guinea [34], Niger [48,75], Lubumbashi (DRC) [23], Gambia [48], and Nigeria [32] showed that children living in households with improved drinking water were less likely to suffer from chronic malnutrition than those from households without access to a safe source of drinking water. Furthermore, three studies found that children living in households with access to safe toilets had a lower risk of chronic malnutrition than children without safe toilets [49,62,75]. Similarly, children whose faeces are properly managed are less likely to be stunted [34]. Finally, three studies have documented that urban children are less likely to suffer from chronic malnutrition than rural children [24,40,74]. However, non-significant effects with regards to the place of residence have been reported in Senegal and Burkina Faso [20,47].

**Community characteristics associated with childhood chronic malnutrition:** community characteristics that were statistically associated with child chronic malnutrition are described in three studies. In Senegal, authors have established that an increase in the percentage of women in the district having decision-making power is statistically associated with reduced childhood chronic malnutrition [20]. In Nigeria, children who came from communities with a low literacy rate were significantly more susceptible to suffer from chronic malnutrition [87]. Finally, researchers in Burkina Faso and Ghana have demonstrated that increasing the immunization coverage of children in a geographical area was associated with a reduction in the prevalence of chronic malnutrition [29,69].

## Discussion

Our discussion first focuses on the methodological aspects of the selected studies. Secondly, it assesses the factors related to childhood chronic malnutrition identified in the selected articles.

**Methods used:** nearly half of the data collected and analyzed in the studies were from the Demographic and Health/Multiple Indicator Cluster Surveys (DHS/MICS) that provide representative results at the national and regional levels. These surveys use a standardized data collection methodology that ensures the comparability of findings across the time horizon and geographic area.

However, there are weaknesses related to the type of data collected. Almost all (63/68) of the study designs are cross-sectional. Thus, the data collected is not well adapted to examining the effects of ephemeral events such as episodes of disease in children on the onset of chronic malnutrition. Indeed, cross-sectional data do not ensure that the occurrence of the event (e.g. episode of diarrhoea) has preceded the resulting outcome (chronic malnutrition). Therefore, it is difficult to prove that the associations made are not by chance. This is particularly problematic in a condition such as chronic malnutrition, where predisposing damaging factors are slowly and permanently established [54]. Given that some of the factors (diarrhoea, fever) are ephemeral, longitudinal data should have been collected and analyzed to assess their effects on chronic malnutrition in children.

Children's eating practices change dramatically in the first five years of life: from zero to six months, they are almost exclusively breastfed; from 6-23 months, they consume breast milk and light foods; and from 24 months, they share the family meal. Consequently, the factors associated with optimal feeding, a close determinant of chronic malnutrition, may differ according to their age. There is evidence to suggest that there are age-specific factors of malnutrition, as demonstrated by authors [34]. Nevertheless, more than two-thirds of the selected studies did not consider this heterogeneity by analyzing the factors of chronic malnutrition in children aged 0-59 months or 6-59 months as homogeneous groups. Future studies should conduct separate analyses using more discrete age groupings in infants and children (e.g. 0-5 months, 6-23 months, and 24-59 months) in order to highlight the specific determinants in each age group.

Finally, it is important to note that 90% of the studies didn´t use a multilevel model although the theoretical hierarchical structure of malnutrition factors [88,89] argues for this model. The search for contextual factors by using a multilevel model would provide a better understanding of children chronic malnutrition in West and Central Africa.

**Associated factors identified:** the review of the articles demonstrated that children's characteristics and maternal characteristics are those most frequently associated with chronic malnutrition. This predominance of child and mother-related factors underlines the importance of these proximate determinants in predicting child chronic malnutrition. Our discussion will address the vulnerability of children to disease, maternal education, the purchasing power of parents, and the decision-making autonomy of mothers, which are the main determinants of chronic malnutrition in children highlighted in West and Central Africa.

**Children's vulnerability to disease:** childhood illness is a major contributing factor to chronic malnutrition. Eleven studies describe a statistical association between the occurrence of diarrhoea, fever or anemia and the onset of chronic malnutrition [20,26,34,40,48,53,56,57,62,77,81, 86]. By affecting nutritional intake, the illness caused stunted growth. For example, when a child has diarrhoea, a frequent symptom in sick children, a good part of the food intake is eliminated through the stools. The body then draws on its reserves to ensure its functioning, which leads to rapid weight loss. In the days following the remission of the diarrhoea, the nutritional intake is primarily allocated by the body to restore the weight deficit. During this time, the child's growth stagnates, which eventually leads to stunted growth [90]. Most other childhood illnesses are associated with digestive disorders such as anorexia, nausea, and vomiting, creating a deficit in food intake, again resulting in stagnation of growth [62]. For example, researchers have established that a child shows an average growth retardation of 0.002 mm per day during an episode of malaria, due in part to digestive disorders [91]. Although childhood illnesses may affect growth due to dietary intake disturbances, this requires frequent or prolonged bouts of illness [54]. In contrast, some research has shown that recent illness (two weeks) may affect children's growth. Thus, future studies should use longitudinal data to better estimate the effects of episodes of illness on chronic malnutrition in children.

The association between chronic malnutrition and children´s characteristics such as age, sex, twinship, birth size, and vaccination status is related (in part) that these factors (e.g. age, sex, twinship, birth size, and vaccination status) make children vulnerable to disease. Authors have demonstrated that the greater susceptibility of twins to malnutrition is due to a higher frequency of congenital malformations, low birth weight, and cerebral palsy, rendering them more susceptible to disease [24]. Similarly, the increased risk of chronic malnutrition in boys is partly related to a higher frequency of premature births (a major factor of ill health) among males compared to females, or to a higher frequency of disease in boys in infancy [20,62,92]. The frequency of twinship or preterm birth in small birth size children explained their increased risk of malnutrition [93].

The effects of age on chronic malnutrition also express a vulnerability to childhood diseases. Indeed, the risk of chronic malnutrition is increased between 6 and 23 months of age when the child's immune system fails due to the decrease in maternal antibodies [87] and due to an as-yet immature immune system. This immune deficiency makes the child particularly vulnerable to disease, which has an impact on his growth. Similarly, routine immunization of children as part of the Expanded Programme on Immunization (EPI) protects them against major childhood diseases. As a result, vaccinated children are relatively less sick and less likely to suffer from chronic malnutrition [32,48,51,58,69,73,86].

In sum, a greater vulnerability to disease in boys, twins, children born with short stature, and unvaccinated children predisposes them to a greater risk of chronic malnutrition. Given that some of these vulnerability factors such as sex, age, twinship, and birth size are non-modifiable factors, research should be directed toward mitigating the effect of those factors that are most commonly associated with chronic malnutrition in children.

**Maternal education:** maternal education is commonly associated with chronic malnutrition in children. Its effect has been highlighted in 19 studies, showing a reduction in the risk of chronic malnutrition when the mothers' education level increases. At school, lessons about nursing, biology, food hygiene, enable mothers and caregivers to acquire useful knowledge to care for and feed babies. In addition, educated people have access to more diversified sources of information (newspapers, posters, etc.), which can increase their knowledge. Thus, capitalizing on their schooling and better access to information, educated mothers have higher knowledge of nutrition [45]. Consequently, their children are less likely to suffer from chronic malnutrition than those of uneducated mothers.

The effect of education can also be mediated through a close-to-home diffusion within communities. Uneducated mothers can learn about good hygiene, care, and feeding practices from their educated counterparts, enabling them to overcome their skill deficits. This neighbourhood effect is often so strong that it leads to the elimination of differences in children's nutritional status caused by the individual educational level of their mothers. This could explain why, in communities where the majority of women are educated, maternal education is not a discriminating factor of children's chronic malnutrition [39,72]. Since the examination of this neighbourhood effect of mothers´ education was considered in only one study [86], future research should investigate its relative contribution to chronic malnutrition.

**Purchasing power of parents:** purchasing power refers to the ability of parents to financially acquire the goods and services necessary for the appropriate growth of children. Some characteristics, such as the mother's employment and income, and the household's economic well-being reflect this effect of parental purchasing power. Women with higher incomes have greater access to food and health services, which is conducive to growth in their children [21,57,67,80]. Similarly, wealthy households have relatively higher incomes that can be used to ensure the successful growth of their children. There is evidence that the purchasing power of mothers has the greatest effect on children's chronic malnutrition, more than that of the father or the whole household [59,64,66,86]. This is explained by the fact that females allocate a larger part of their income to food expenditures [94].

Despite this, some studies have found no effect of purchasing power estimated by the household income level on childhood chronic malnutrition [39,59,64,66,70,86]. This counter-intuitive result may be linked to limitations in the definition of the indicator of purchasing power. In most studies, household income is assessed based on a synthetic index made up of housing characteristics and amenities of the household [95]. This indicator of wealth/poverty may not adequately reflect the true economic status of the household members. Residents of households classified as rich according to this indicator may be poor and vice versa. Moreover, this indicator of relative poverty only assesses differences between extreme classes of socioeconomic levels [96]. Due to these limitations, the use of this indicator to analyze the effects of parental purchasing power on child chronic malnutrition is imperfect.

To be complete, the assessment of parental purchasing power on children's nutritional status must take into account the community income level, as access to goods and services is dependent on it. In fact, food and healthcare are relatively more expensive in upper-income communities found in large urban centers [97,98]. Thus, at equal income levels, people living in an upper-income community may have more limited access to healthcare and food, compared to their peers living in a lower-income community. It is, therefore, appropriate to consider the context of relative wealth in determining the effects of parental income on chronic child malnutrition.

**Maternal autonomy:** this concept refers to “a woman's ability to influence decisions about family, finances, and spending, work, social outings, health care, travel, family planning, and child care...” [99]. The effects of maternal leadership, participation in decision making, and experience and attitude toward violence highlighted in the selected studies reflect the impact of autonomy [33,41,72,79,84]. For example, a woman who has some leadership can work to increase the share of household resources devoted to food, since they devote a larger share of their income for this purpose [94]. Similarly, a mother who has greater freedom of movement can more easily travel to the health clinic to receive care and advice, which is beneficial for the proper growth of her children. Similarly, women who do not fear violence from their husbands are more likely to follow the nutritional advice they receive than those who do fear them.

In summary, the autonomy of mothers to make decisions concerning the care and feeding of their children is an important determining factor, especially in the contexts of child care and feeding which are almost exclusively their responsibility. Studies on chronic malnutrition should take into account the mothers´ autonomy whose effects have not yet been sufficiently analyzed in West and Central Africa.

## Conclusion

This systematic search and review of the scientific literature have identified child, maternal, household, and community characteristics that are associated with chronic malnutrition in children living in West and Central Africa. Despite the relative abundance of scientific evidence on the subject, the effects of some major factors such as children's vulnerability to disease, maternal education, and maternal autonomy are still poorly defined. Future studies should focus on a deeper investigation of the effects of these factors. In addition, the use of subgroups in analyzing age, and longitudinal data, would provide complete view of childhood chronic malnutrition factors in West and Central Africa.

### What is known about this topic

- West and Central Africa, which is the most affected sub-region;
- Factors contributing to childhood chronic malnutrition are studied in several empirical studies.

### What this study adds

- It is the first knowledge synthesis of factors associated with childhood stunting in West and Central Africa;
- It shows that there would be a contextual effect of mother's education and purchasing power that must be taken into account in future studies;
- Child´s vulnerability to disease is a factor of childhood chronic malnutrition.
